# The role of costing in the introduction and scale‐up of HIV pre‐exposure prophylaxis: evidence from integrating PrEP into routine maternal and child health and family planning clinics in western Kenya

**DOI:** 10.1002/jia2.25296

**Published:** 2019-07-22

**Authors:** D Allen Roberts, Ruanne V Barnabas, Felix Abuna, Harison Lagat, John Kinuthia, Jillian Pintye, Aaron F Bochner, Steven Forsythe, Gabriela B Gomez, Jared M Baeten, Grace John‐Stewart, Carol Levin

**Affiliations:** ^1^ Department of Epidemiology University of Washington Seattle WA USA; ^2^ Department of Global Health University of Washington Seattle WA USA; ^3^ Department of Medicine University of Washington Seattle WA USA; ^4^ University of Washington in Kenya Nairobi Kenya; ^5^ Kenyatta National Hospital Nairobi Kenya; ^6^ Avenir Health Glastonbury CT USA; ^7^ Department of Global Health and Development London School of Tropical Hygiene and Medicine London United Kingdom

**Keywords:** HIV, PrEP, women, prevention, cost‐effectiveness, health economics, cost analysis

## Abstract

**Introduction:**

Understanding the cost of strategies to reach and deliver pre‐exposure prophylaxis (PrEP) to priority populations is essential to assess the cost‐effectiveness and budget impact of HIV prevention programmes. Providing PrEP through maternal and child health and family planning clinics offers a promising strategy to reach women in high HIV burden settings. We estimated incremental costs and explored the cost drivers of integrating PrEP delivery into routine maternal and child health and family planning services in Kenya.

**Methods:**

We conducted a costing study from the provider perspective within the PrEP Implementation for Young Women and Adolescents programme in western Kenya. We identified all within‐ and above‐facility activities supporting PrEP delivery and measured clinical service time using time‐and‐motion studies. We obtained input costs from programme budgets, expenditure records and staff interviews. We estimated changes in costs if creatinine testing were postponed from initiation to first follow‐up visit and if PrEP were prioritized to clients at high HIV risk using a behavioural risk assessment tool. We also projected costs under Ministry of Health (MOH) implementation assuming MOH salaries and programme supervision. We estimated annual numbers of PrEP visits from programme data abstracted from 16 facilities between November 2017 and June 2018. We report the cost per client‐month of PrEP dispensed in 2017 USD.

**Results:**

For an annual programme output of 24,005 screenings, 4198 PrEP initiations and 4427 follow‐up visits, the average cost per client‐month of PrEP dispensed in the study was $26.52. Personnel, drugs and laboratory tests comprised 43%, 25% and 14% of programme costs respectively. Postponing creatinine testing and prioritizing PrEP delivery to clients at high HIV risk reduced total programme costs by 8% and 14% respectively. In the MOH scenario assuming no changes in outputs, the projected cost per client‐month of PrEP dispensed decreased to $16.54 and total programme costs decreased by 38%.

**Conclusions:**

Incremental PrEP costs are sensitive to the service delivery strategy used to engage priority populations. Postponing creatinine testing and prioritizing PrEP delivery to clients at high HIV risk may reduce costs. Context‐specific cost data are crucial to assess the cost‐effectiveness and affordability of PrEP delivery models.

## 1 Introduction

Despite remarkable progress in expanding access to antiretroviral therapy (ART), an estimated 200,000 women aged 15 to 24 in sub‐Saharan Africa were newly infected with HIV in 2017 1. Pre‐exposure prophylaxis (PrEP) prevents HIV infection and offers promise as a female‐controlled HIV prevention strategy 2, 3. Effective models for PrEP delivery to young women are needed to maximize population‐level benefits. Budgets have competing demands, and evidence on the cost, affordability and potential impact of PrEP programmes is necessary to guide policy decisions about the choice and implementation of prevention interventions 4.

PrEP programmes that achieve widespread coverage and adherence among individuals at high risk of acquiring HIV infection (priority populations) will maximize population‐level impact 5, 6, 7, 8, 9, 10, 11. However, the cost and yield of engaging priority populations will vary across settings, affecting cost‐effectiveness and budget impact conclusions 12. While several studies have projected the potential cost‐effectiveness of PrEP delivery in sub‐Saharan Africa, few service delivery models for identifying and providing PrEP to priority populations have been defined that could substantiate the costs assumed in modelling studies 13, 14, 15, 16, 17, 18. Primary costing studies of PrEP delivery are sparse, and research describing how costs vary across outreach and service delivery strategies is limited 19, 20, 21.

Integrating PrEP into other medical and nonmedical services may be an efficient strategy for reaching priority populations. For example, offering PrEP to women through maternal and child health (MCH) or family planning (FP) programmes may have low incremental costs. Pregnant and postpartum women in sub‐Saharan Africa have high HIV incidence, and recent evidence suggests that HIV risk may be elevated in pregnancy and postpartum periods 22, 23, 24. However, no prior studies have estimated the cost of delivering PrEP to women through MCH and FP clinics. A previous modelling study of PrEP administration to pregnant and breastfeeding women varied PrEP programme costs per patient‐year from $80 to $720 per year, reflecting large uncertainty in the absence of data 25.

We present the results from a costing study for the PrEP Implementation for Young Women and Adolescents (PrIYA) programme, an implementation project delivering PrEP in 16 MCH and FP clinics in western Kenya. We estimated the incremental cost of integrating PrEP delivery into routine MCH and FP services. Furthermore, we explored the cost implications of service delivery modifications such as timing of creatinine monitoring and prioritized delivery to women identified as having high risk for HIV infection.

## 2 Methods

### 2.1 Study setting

The PrIYA programme is an implementation project to evaluate PrEP delivery strategies to young women through MCH and FP clinics in Kenya 26. PrIYA is part of the DREAMS Innovation Challenge funded by the U.S. President's Emergency Plan for AIDS Relief (PEPFAR) and managed by JSI Research & Training Institute, Inc. In collaboration with Department of Health and Sanitation, Kisumu County and the Kenya National AIDS and STI Control Programme (NASCOP), PrIYA has been implemented in 16 facilities (nine public hospitals, four mission hospitals, one private hospital, one health centre and one dispensary) in Kisumu County and involves centralized supervision and administration by programme staff. This region has an estimated adult HIV prevalence of 16% and high incidence of HIV among pregnant women 27, 28. Women attending MCH and FP clinics are screened by nurses for behavioural risk factors for HIV and willingness to consider PrEP. All medically eligible (HIV‐negative and creatinine clearance <50 mL/min by national guidelines) women who are interested in PrEP are offered same‐day PrEP initiation and can return to the same clinic for monthly follow‐up visits and refills 29. PrEP is delivered either by the same nurse providing routine MCH and FP services or by a separate nurse in an adjoining room. Nurses perform point‐of‐care creatinine testing at initiation and dispense medication directly to clients. Additional programme details have been described elsewhere 30. The PrIYA protocol was approved by the University of Washington Human Subjects Division and the Kenyatta National Hospital/University of Nairobi Ethical Review Committee. Participants provided oral informed consent.

### 2.2 Costs

We estimated the incremental economic cost of PrEP delivery from the provider perspective following the principles outlined in the Global Health Cost Consortium Reference Case 31. We categorized costs as either fixed (constant irrespective of programme output over the course of one year) or variable (costs directly related to programme output). To estimate variable costs, we measured resource use at a sample of eight facilities representative of clinic size, ownership (public, mission or private), and type (MCH vs FP). For each PrEP clinical activity (behavioural screening and counselling, initiation, and follow‐up visits), we measured the cost of drugs, clinical personnel, laboratory testing and other supplies. We estimated clinical personnel unit costs using time‐and‐motion studies and multiplying the average time spent in each activity (in minutes) by the cost per minute (including both salary and benefits). For supplies and commodity costs, we observed resource use for each activity and multiplied the relevant quantity by input costs obtained from programme budgets or centralized price lists. Drug costs for oral co‐formulated tenofovir disoproxil fumarate/emtricitabine ($6.75 per 30 days) included the cost of purchase from the manufacturer as well as central storage and distribution costs. Fixed costs included centralized start‐up costs (microplanning and training), capital (equipment, furniture), overheads (e.g. building costs, transportation and airtime) and administrative and supervisory personnel supporting PrEP delivery. We annualized start‐up and capital costs over the expected useful life (assumed to be five years or fewer) using a discount rate of 3% 31. We allocated building space based on the proportion of all MCH or FP visits that included a PrEP encounter. We multiplied the average size of the room in which PrEP screening and initiation were conducted by a rental rate estimated from nearby commercial properties. This analysis excludes the cost of any research activities that would not be part of routine PrEP service delivery.

We calculated programme‐level average unit costs for each clinical activity (screening, initiation and follow‐up visits) by allocating fixed costs to each activity and adding the activity's average variable cost. Fixed costs that could not be assigned exclusively to a single activity were apportioned using hourly rate allocation based on clinical service time 32. We adjusted all costs to 2017 currency using GDP deflators and converted to US dollars (USD) using the 2017 average exchange rate (1 USD = KSh 103.40) 33. We analysed costs in Excel 2018 (Microsoft, Redmond, USA). Additional details about the costing methodology, including the Excel file used for the analysis, are available in the Supporting Information.

### 2.3 Programme volume

We used data collected as part of routine monitoring to estimate the numbers of women screened, initiated and dispensed PrEP over a one‐year period. Study staff abstracted standardized client records in all 16 facilities from 20 November 2017 to 15 June 2018. We extrapolated programme volume to one year assuming no changes in the pattern of visits. We analysed programme volume using R version 3.5.1 34. Further details are available in the Supporting Information.

### 2.4 Cost metrics

We estimated the total programme cost (across 16 facilities) by multiplying the number of screening, initiation and follow‐up visits by their respective average unit costs and summing the total. We then calculated the cost per client‐month of PrEP dispensed as follows:

$$\begin{array}{c} \mathrm{Cost}\;\mathrm{per}\;\mathrm{client}-\mathrm{month}\;\mathrm{of}\;\mathrm{PrEP}\;\mathrm{dispensed}\\ =\frac{\text{Total programme cost}}{\#\text{months of PrEP dispensed}} \end{array}$$

### 2.5 Scenarios

To evaluate the potential cost ramifications of different delivery scenarios, we projected costs incurred under the following conditions: (1) postponing creatinine testing to the first follow‐up visit rather than initiation; (2) restricting PrEP initiation to clients identified as having high risk for acquiring HIV; and (3) if the programme were entirely implemented through the Ministry of Health (MOH).

The first scenario (postponing creatinine testing) is motivated both by the low prevalence (8/4007) of ineligible creatinine tests among PrIYA clients at initiation as well as the considerable proportion of clients who choose to discontinue PrEP within a month after initiating. This scenario is expected to decrease programme costs by reducing the number of creatinine tests conducted. For the second scenario, we categorized clients as having high risk of HIV infection based on reporting at least one of the following risk factors assessed at their first PrEP screening visit: (1) current partner with unknown or positive HIV status; (2) positive rapid plasma regain syphilis test; or (3) reporting at least one of the following in the prior six months: (a) exchanging sex for money or other favours; (b) diagnosis or treatment for a sexually transmitted infection; (c) forced to have sex against will; (d) experiencing intimate partner violence; (e) sharing needles while engaging in injection drug use; or (f) using post‐exposure prophylaxis more than twice. Assessment of these specific risk factors for PrEP consideration is recommended as part of national guidelines 29. This scenario is expected to decrease total programme costs by reducing the number of clients who initiate and continue on PrEP.

In the third scenario, we revised costs to reflect a programme implemented entirely through the MOH. First, we adjusted clinical personnel salaries to reflect government cadre‐specific salary scales (including benefits). Second, we estimated the cost of facility, sub‐county and county supervisory activities that are planned to subsume PrIYA administrative staff responsibilities. Last, we replaced the cost of the point‐of‐care assay used in PrIYA with the average facility price for creatinine testing. These projections assume no changes in programme output. We conducted a sensitivity analysis under the MOH scenario to explore how costs might change with varying uptake and retention, assuming constant fixed costs.

## 3 Results

### 3.1 Overall programme costs and unit costs

For an annual programme output of 24,005 screenings, 4198 PrEP initiations, and 4427 follow‐up visits, the estimated total annual programme cost as implemented was $204,253 (Table 1). Personnel (43%), drugs (25%) and laboratory tests (14%) comprised the largest cost categories. Supervision and administration accounted for nearly two‐thirds of personnel costs. The average cost per client‐month of PrEP dispensed was $26.52. The unit costs of PrEP screening, initiation and follow‐up encounters were $2.91, $19.18 and $12.16 respectively (Table 2).

**Table 1:** Total programme cost and average cost per client‐month of PrEP dispensed (2017 USD)

	Total annual cost (USD)	Average cost per client‐month of PrEP dispensed (USD)
Variable		
Personnel (clinical)	37,535	4.87
Drugs	51,997	6.75
Laboratory testing	27,830	3.61
Other supplies	3,616	0.47
Sub‐total	120,978	15.71
Fixed		
Microplanning	1,366	0.18
Training	2,898	0.38
Personnel (supervision and administration)	50,924	6.61
Capital (e.g. creatinine machines, furniture)	3,925	0.51
Overhead (e.g. building, airtime, transportation)	24,162	3.14
Sub‐total	83,275	10.81
Summary	204,253	26.52

**Table 2:** Unit cost breakdown by clinical activity (2017 USD)

	Screening	Initiation	Follow‐up^a^
Variable unit cost			
Personnel (clinical)	0.91	1.47	2.14
Drugs	0.00	6.75	5.34
Laboratory testing	0.00	5.76	0.83
Other supplies	0.02	0.32	0.41
Sub‐total	0.93	14.30	8.71
Fixed unit cost	1.98	4.88	3.45
Total unit cost (variable + fixed)	2.91	19.18	12.16
Number	24,005	4,198	4,427
Total annual cost	69,876	80,525	53,852

a Follow‐up unit costs are weighted averages of the costs of visits with (79%) and without (21%) PrEP dispensation.

### 3.2 Cost implications of service delivery modifications

Table 3 shows the estimated impact of postponing creatinine testing to the first follow‐up visit or prioritizing PrEP initiation to clients at high risk of HIV infection on the total programme cost and cost per month of PrEP dispensed. Postponing creatinine testing would reduce the annual number of tests by two‐thirds (from 4198 to 1370) and decreases estimated programme costs by 7.5%, resulting in a cost of $24.53 per client‐month of PrEP dispensed. Clients at high risk for HIV infection (at least one baseline risk factor) accounted for 34% of screening encounters but 68% of PrEP initiations. The most common risk factor was having a partner of unknown status (89% of clients with at least one baseline risk factor). Restricting PrEP initiation to clients at high risk of HIV lowered total programme costs by 14%. Under this scenario, the cost per client‐month of PrEP dispensed only to clients with high risk of HIV infection was $31.88.

**Table 3:** Estimated cost implications of service delivery modifications

Scenario	Total annual cost (USD)	Cost per client‐month of PrEP dispensed (USD)
As implemented	204,253	26.52
Postponed creatinine^a^	188,932	24.53
Prioritized delivery to clients at high risk for HIV infection^b^	175,793	31.88^c^

^a^Creatinine testing postponed from initiation to first follow‐up visit; ^b^High risk is defined as having at least one of the following risk factors at baseline: Current partner with unknown or positive HIV status, positive rapid plasma reagin syphilis test, or reporting at least one of the following in the prior six months: exchanging sex for money or other favours, diagnosis or treatment for a sexually transmitted infection, forced to have sex against will, experiencing intimate partner violence (IPV), sharing needles while engaging in injection drug use, using post‐exposure prophylaxis more than twice; ^c^Unit cost is calculated by dividing the total programme cost by the number of person‐months of PrEP dispensed to clients at high risk of HIV infection.

### 3.3 Projected costs under MOH implementation

**Figure 1:**
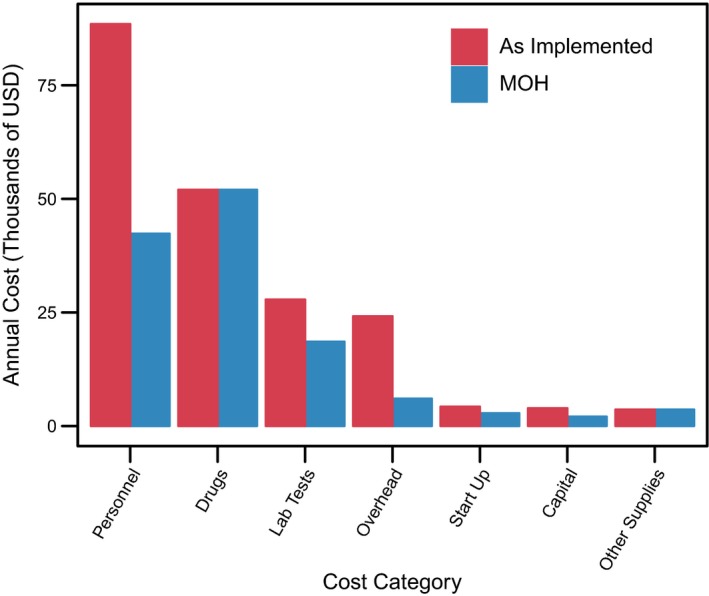
Total annual programme cost (2017 USD) by category as implemented and in the Ministry of Health (MOH) scenario. The MOH scenario assumes public sector clinical staff salaries instead of study salaries; study administrative staff responsibilities are subsumed into routine facility, sub‐county, and county supervision; and facility‐based creatinine testing instead of point‐of‐care.

We projected how programme costs might change under Kisumu County MOH implementation assuming no changes in outputs. Substituting public‐sector clinical staff salaries for PrIYA nurse salaries decreased the cost per client‐month of PrEP dispensed from $26.52 to $25.92 (Table 4). Using the estimated cost of planned MOH supervision in place of PrIYA administration lowered the cost per client‐month of PrEP dispensed from $25.92 to $18.00. Replacing the cost of the point‐of‐care creatinine assay with facility creatinine prices further decreased the cost per client‐month of PrEP dispensed to $16.54. Overall, we estimated the total programme cost under the MOH scenario to be $127,421, which constitutes a 38% decrease compared to PrIYA implementation, and the average cost per client‐month of PrEP dispensed decreased to $16.54. The largest cost components were drugs (41%), personnel (33%) and laboratory tests (15%) (Figure 1 and Figure S3). Overall personnel costs were 52% lower under the assumption that project coordinator staff activities would be subsumed under existing facility and above‐facility supervisory structures.

**Table 4:** Cost projections under Ministry of Health (MOH) implementation assuming constant output

Scenario	Total annual cost (USD)	Cost per client‐month of PrEP dispensed (USD)
As implemented	204,253	26.52
With public‐sector clinical staff salaries	199,613	25.92
With MOH supervision^a^ and public‐sector clinical staff salaries	138,609	18.00
With facility creatinine testing^b^, MOH supervision^a^, and public‐sector clinical staff salaries	127,421	16.54

^a^PrIYA administrative staff responsibilities are subsumed into routine facility, sub‐county and county supervision; ^b^Using prices for facility‐based creatinine testing instead of a point‐of‐care assay.

We evaluated the sensitivity of costs under the MOH scenario to assumptions about programme output. Doubling both the proportion of screening encounters that result in an initiation (from 17% as observed to 34%) and the average number of follow‐up visits within a year among clients with at least one follow‐up visit (from 2.6 as observed to 5) increases estimated programme costs by 224% and lowers the cost per client‐month of PrEP dispensed to $12.96. In comparison, halving both uptake and retention increases the cost per client‐month of PrEP dispensed to $25.31 while reducing total programme costs by 42%.

## 4 Discussion

We explored the relationship between costs and service delivery strategies using primary data from an implementation study of integrating PrEP into MCH and FP clinics, as part of the DREAMS Innovation Challenge funded by PEPFAR. Offering PrEP to women through MCH and FP clinics as done in this study would cost on average $26.52 per client‐month of PrEP dispensed, with personnel and drugs accounting for 43% and 25% of programme costs respectively. In comparison, an analysis of PrEP delivery to female sex workers and men who have sex with men (MSM) in Nairobi that found that drugs accounted for 15‐19% of total costs 20. The Nairobi study estimated higher unit costs ($33 to 44 per client‐month of PrEP dispensed) than our study despite similar drug unit costs. The difference in the two programme costs may reflect the increased resources used in outreach efforts needed to contact FSW and MSM compared to a clinic‐based strategy that integrated PrEP within existing services. However, our estimated unit costs are higher than analyses among FSW and MSM conducted in South Africa, which estimated costs per client‐month of $17‐18 10, 19. This programme had substantially lower drug costs (<$5 per month) as well as high uptake and retention, both of which contributed to lower unit costs. Additional efforts are needed to better understand differences between delivery strategies and to standardize cost reporting.

Prioritizing PrEP delivery to clients at high risk for HIV infection can reduce total costs if these clients are easily identified. In our study, total programme costs decrease by 14% if initiation occurs only among clients with baseline behavioural risk factors. Clients with baseline risk factors were more likely to initiate PrEP, demonstrating that risk prioritization is to some extent occurring as these clients self‐select to initiate PrEP when universally offered 26. Eighty‐nine percent of clients with a baseline risk factor had a partner with unknown HIV status, highlighting that increasing partner testing could improve client risk assessment. Given the low cost of HIV self‐test kits ($2 as negotiated by the Bill and Melinda Gates Foundation), providing HIV self‐test kits to promote partner testing might be an efficient method for refining client decisions about PrEP 35, 36. The utility of these strategies will depend on the how well risk can be evaluated by both client and provider. An ongoing randomized trial using an HIV risk assessment tool designed for peripartum women and self‐testing to guide PrEP delivery among pregnant women will help evaluate the potential impact of this strategy 37, 38. In the process of risk assessment, it is also important that PrEP delivery programmes do not stigmatize women or suggest they are the primary population responsible for HIV prevention. Validated and context‐specific risk assessment tools for a range of populations, including men, are needed to guide prevention programmes.

Creatinine testing consumed significant resources, and previous studies of reducing the frequency of kidney function monitoring have not shown harm 39. Due to high numbers of clients discontinuing PrEP after initiation, deferral of creatinine testing from initiation to the first follow‐up visit would save an estimated 7.5% of overall programme costs. Notably, only 0.2% of clients in the programme had creatinine clearance measured at less than 50 mL/min at initiation (the NASCOP threshold for PrEP ineligibility). Postponing creatinine testing by one month does not present a major departure from Kenya national guidelines, which recommend baseline and then annual testing but permit PrEP delivery without testing if laboratory facilities are unavailable 29.

Implementation projects are essential for demonstrating the impact and costs of strategies for introducing PrEP to at‐risk populations; however, their costs may not reflect typical MOH settings. Our analysis projects overall programme costs could decrease by 38% under routine MOH implementation. This large potential cost reduction is consistent with previous PrEP costing studies that have compared observed costs to projections in an MOH scenario. A demonstration project of PrEP as a bridge to ART among serodiscordant couples in Uganda reported estimated unit costs of $408 (as studied) and $92 (MOH scenario) per couple per year 40. However, the projected costs in the MOH scenario were highly sensitive to assumptions about programme volume. The degree to which unit costs will change will depend on how programme output is affected by changes in staff and supervision.

Within a facility‐based setting, additional service delivery modifications may affect PrEP costs. For example, providing multiple months of PrEP prescription for established clients could improve retention by requiring fewer visits, as has been demonstrated in some ART programmes 41, 42. Additionally, task shifting PrEP screening counselling to HTS counsellors may reduce costs and alleviate nurse time burden. HIV testing provides a natural entry point for discussions about HIV prevention and PrEP use, as PrEP initiation is contingent upon a negative test and behavioural assessment. While task shifting HIV services has demonstrated efficiency gains across a wide variety of settings 43, 44, it is possible that programme output would be affected. Implementation studies are needed to evaluate the utility of these models.

Our analysis has several limitations. The baseline behavioural risk factors used to classify clients are not based on a validated risk score and may not fully capture HIV risk. The unit cost of client‐month of PrEP dispensed reflects neither the client's true HIV risk nor drug adherence, both of which are crucial parameters for cost‐effectiveness studies. In addition, our assumption of constant volume with reduced costs under MOH implementation may be unrealistic. Programme output may decrease without PrIYA clinical staff and programme support. Alternatively, PrEP uptake and retention may increase over time with expanded community awareness and sensitization, and counselling time may decrease as clients become more familiar with the intervention. Improving demand generation, messaging and support strategies will be critical to increasing PrEP usage in this population 3, 45. We did not address whether additional MOH clinical staff would be required to support PrEP initiation, which would add human resource costs. Last, the 16 facilities involved in PrIYA were primarily hospitals in urban and semi‐urban environments that may benefit from economies of scale. These facilities may not be representative of all MCH and FP clinics in western Kenya, so caution must be taken in generalizing these cost estimates to other settings. We were not able to address facility‐level variation in PrEP service delivery costs given the centralized implementation of the programme; further research under routine conditions is needed to identify facility‐level drivers of service delivery costs.

Despite these limitations, cost projections are useful to evaluate the relative impact that programme modifications may have on budgets. Benefits of offering PrEP through MCH and FP services include potential economies of scope and convenient access to a priority population, without ancillary outreach activities to needed reach them. However, the degree to which women perceive themselves at risk and choose to take PrEP as part of routine MCH and FP services will critically affect costs, coverage and impact. While our study focused on a PrEP delivery strategy for women, ongoing cost data collection efforts nested in implementation science evaluations are needed to provide up‐to‐date evidence on the costs of delivery strategies to reach other priority populations, including adolescents, serodiscordant couples, FSW and MSM. Such data will be critical for understanding the potential success of PrEP programmes at the country level and will serve as invaluable inputs to mathematical models that aim to produce more accurate estimates of potential cost‐effectiveness.

## 5 Conclusions

MCH and FP services offer a potential PrEP delivery platform to efficiently reach large numbers of at‐risk women in high HIV burden settings. Postponing creatinine testing and prioritizing PrEP delivery to clients at high HIV risk are potential strategies to reduce costs. Sub‐population‐specific costing studies are needed to evaluate the costs of delivering PrEP to priority populations in other settings. Cost‐effectiveness studies of PrEP scale‐up need context‐specific costing data in order to accurately inform policy.

## Competing interests

The authors have no competing interests to declare.

## Authors’ contribution

DAR, CL and RVB designed and conducted the study with contributions from FA, HL, JK, JP, AB, JMB and GJS. GBG and SF provided insight into the interpretation of the results and assisted with writing the manuscript. DAR wrote the first draft of the manuscript. All authors have read and approved the final manuscript.

## Supporting information


**Figure S1.** Overview diagram of costing methodology.
**Figure S2.** Map of PrIYA health facilities in Kisumu County, Kenya.
**Figure S3.** Percentage of total programme cost across cost categories as implemented and under Ministry of Health (MOH) scenario.*
**Table S1.** Input costs of key PrEP delivery components (2017 USD)
**Table S2.** Time (minutes) for clinical service delivery components estimated from time‐and‐motion studies
**Table S3.** Total annual programme cost and unit cost per client‐month of PrEP dispensed (2017 USD) in Ministry of Health (MOH) scenario*
**Table S4.** Unit cost breakdown by clinical activity (2017 USD) under Ministry of Health (MOH) scenario*
**Table S5.** 5% Discount rate
**Table S6.** 10% Discount rate
**Table S7.** 15% Discount rate

**Data S1.** Cost Calculations Spreadsheet.
