# Supplementary material for: The role of costing in the introduction and scale‐up of HIV pre‐exposure prophylaxis: evidence from integrating PrEP into routine maternal and child health and family planning clinics in western Kenya
Source: J Int AIDS Soc. 2019 Jul 22;22(Suppl Suppl 4):e25296. doi: 10.1002/jia2.25296 (PMC6643078; doi:10.1002/jia2.25296)
Supplement: Supplementary file 1 — Figure S1. Overview diagram of costing methodology. Figure S2. Map of PrIYA health facilities in Kisumu County, Kenya. Figure S3. Percentage of total programme cost across cost categories as implemented and under Ministry of Health (MOH) scenario.* Table S1. Input costs of key PrEP delivery components (2017 USD) Table S2. Time (minutes) for clinical service delivery components estimated from time‐and‐motion studies Table S3. Total annual programme cost and unit cost per client‐month of PrEP dispensed (2017 USD) in Ministry of Health (MOH) scenario* Table S4. Unit cost breakdown by clinical activity (2017 USD) under Ministry of Health (MOH) scenario* Table S5. 5% Discount rate Table S6. 10% Discount rate Table S7. 15% Discount rate [file JIA2-22-e25296-s001.docx]

**Supporting Information**

**A. Costing Methodology**

**Figure S1: Overview Diagram of Costing Methodology**

**
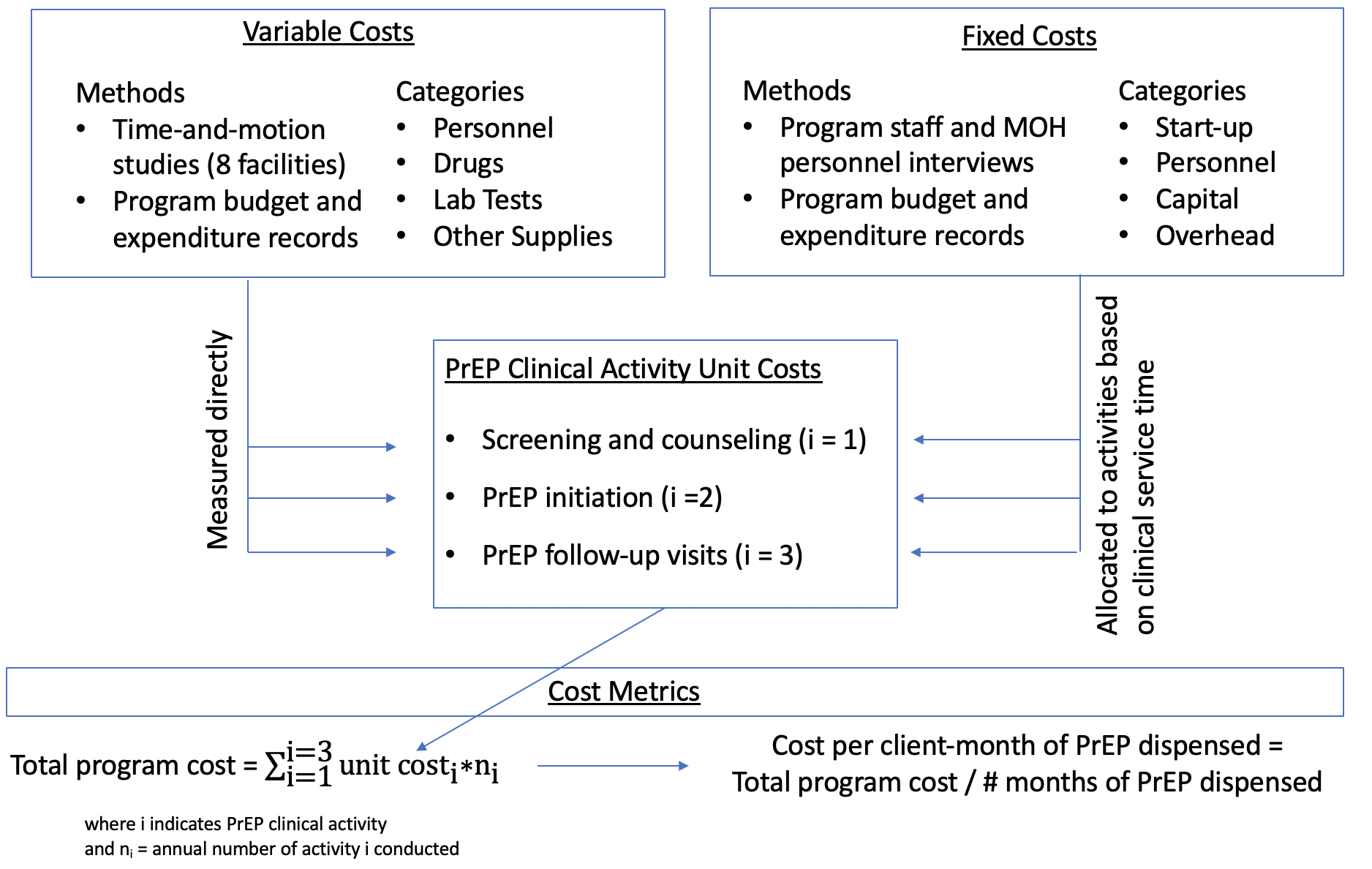
**

**Variable Cost Categories**

1. Estimates of resource use for variable costs were obtained from a sample of eight of the 16 PrIYA facilities.
2. *Personnel:* Included clinical staff costs for PrEP screening, initiation, HIV testing, and follow-up visits Personnel time was estimated via time-and-motion studies. In the MOH scenario, PrIYA nurse salaries were replaced by MOH salaries.
3. *Drugs:* Drug costs for oral co-formulated tenofovir disoproxil fumarate/emtricitabine ($6.75 per 30 days) included the cost of purchase from the manufacturer as well as storage and transportation costs (charged at 8% of the cost of the product).
4. *Laboratory tests:* Included the cost of the Determine HIV test to confirm HIV-negative status for clients at initiation and follow-up visits as well as the cost of point-of-care creatinine test strips. Also included consumable supplies used in test administration (ie, gloves, lancets, etc). In the MOH scenario, the point-of-care creatinine cost was replaced by the average facility charge for creatinine testing across PrIYA facilities.
5. *Other Supplies:* Included printing cost of appointment cards, prescription pads, and PrEP encounter records.

**Fixed Cost Categories**

1. *Start-up (annualized over five years)*
   1. *Microplanning:* Included meetings with county-level officials (ie, technical working group and task force meetings), onboarding meetings with facility personnel, and printing costs to update standardized MOH reporting tools in order to include TDF/FTC.
   2. *Training:* Included staff, venue, and supplies costs for initial nurse trainings as well as commodities and logistics management and information systems (LMIS) facilitation trainings for county and sub-county pharmacists and health records officers.
2. *Personnel*: Included facility staff costs spent on routine reporting, drug accounting, and conducting weekly phone meetings between nurses and program coordinators to debrief on PrEP delivery. Also included the annual salaries and benefits of three PrIYA program staff multiplied by the fraction of their time spent on service delivery management as opposed to research-specific activities (estimated from staff interviews). In the MOH scenario, weekly phone meetings between PrIYA staff were replaced by quarterly PrEP refresher trainings at each facility and PrIYA coordinator salaries were replaced by estimated costs for quarterly supervisory visits from county- and sub-county level health management teams (estimated from interviews with MOH staff).
3. *Capital:* Included the cost of creatinine machines, control solutions, and furniture used during PrEP encounters. Furniture costs were multiplied by the fraction of all MCH and FP encounters recorded at each facility that included PrEP activities. Useful life years assumed to be five years for most items (select items that needed to be replaced yearly, such as creatinine control solutions, were assigned useful life of one year).
4. *Overhead:* Included transportation costs for weekly facility visits conducted by PrIYA program staff, airtime, printing costs for reporting tools, and building and utility costs (estimated using nearby rental properties and multiplied by the fraction of all MCH and FP encounters recorded at each facility that included PrEP activities). Weekly transportation costs were excluded from the MOH scenario under the premise that PrEP supervisory activities would be integrated within existing scheduled visits for PMTCT and reproductive health supervision.

**C. Key input costs**

**Table S1: Input costs of key PrEP delivery components (2017 USD)**

| **Item** | **Cost (2017 USD)** | **Source** |
| --- | --- | --- |
| 30 days TDF/FTC | 6.75^*^ | MOH Personal Communication |
| Point-of-care creatinine test strip^†^ | 4.50 | Project budget |
| Determine HIV test | 0.83 | Project budget |
| Project nurse monthly salary + allowances | 819 | Project budget |
| Facility-based creatinine test^‡^ | 2.51 | PrIYA facility survey |
| MOH nurse monthly salary + allowances | 718 | Central MOH Salary Scale |

^*^Purchase price from manufacturer of $6.25 per 30 day prescription plus 8% storage and distribution cost

^†^XPress StatSensor® Creatinine Meter

^‡^Based on average charge for creatinine test across PrIYA facilities

**D. Time-and-motion studies**

Time-and-motion studies were conducted in a sample of eight of the 16 PrIYA facilities (**Figure S2**). Facilities were selected to be representative of clinic size, ownership (public, mission, or private), and type (MCH vs FP). A trained PrIYA staff member directly observed screening, PrEP initiation, PrEP follow-up visits, and HIV testing and counselling sessions and recorded the clinical provider time spent in each encounter. Time spent on any research-related activities outside the scope of routine PrEP service delivery were excluded. The average encounter time across all observations was multiplied by the hourly personnel cost to estimate personnel costs per clinical encounter. Time-and-motion results are displayed in **Table S2.** In addition to personnel time, input resource usage (e.g., lab tests, consumables, etc) were observed and combined with personnel costs to estimate variable unit costs.

**Table S2: Time (minutes) for clinical service delivery components estimated from time-and-motion studies**

| **Activity** | **Mean** | **Median** | **25^th^ percentile** | **75^th^ percentile** |
| --- | --- | --- | --- | --- |
| Screening | 8 | 7 | 4 | 11 |
| Initiation^†^ | 13 | 11 | 8 | 15 |
| Creatinine | 4 | 3 | 2 | 4 |
| HIV testing and counselling | 13 | 12 | 10 | 15 |
| Follow-up visit | 9 | 9 | 8 | 10 |

^†^Includes completing PrEP medical record, point-of-care creatinine testing, and medication dispensation

**Figure S2: Map of PrIYA Health Facilities in Kisumu County, Kenya**

**
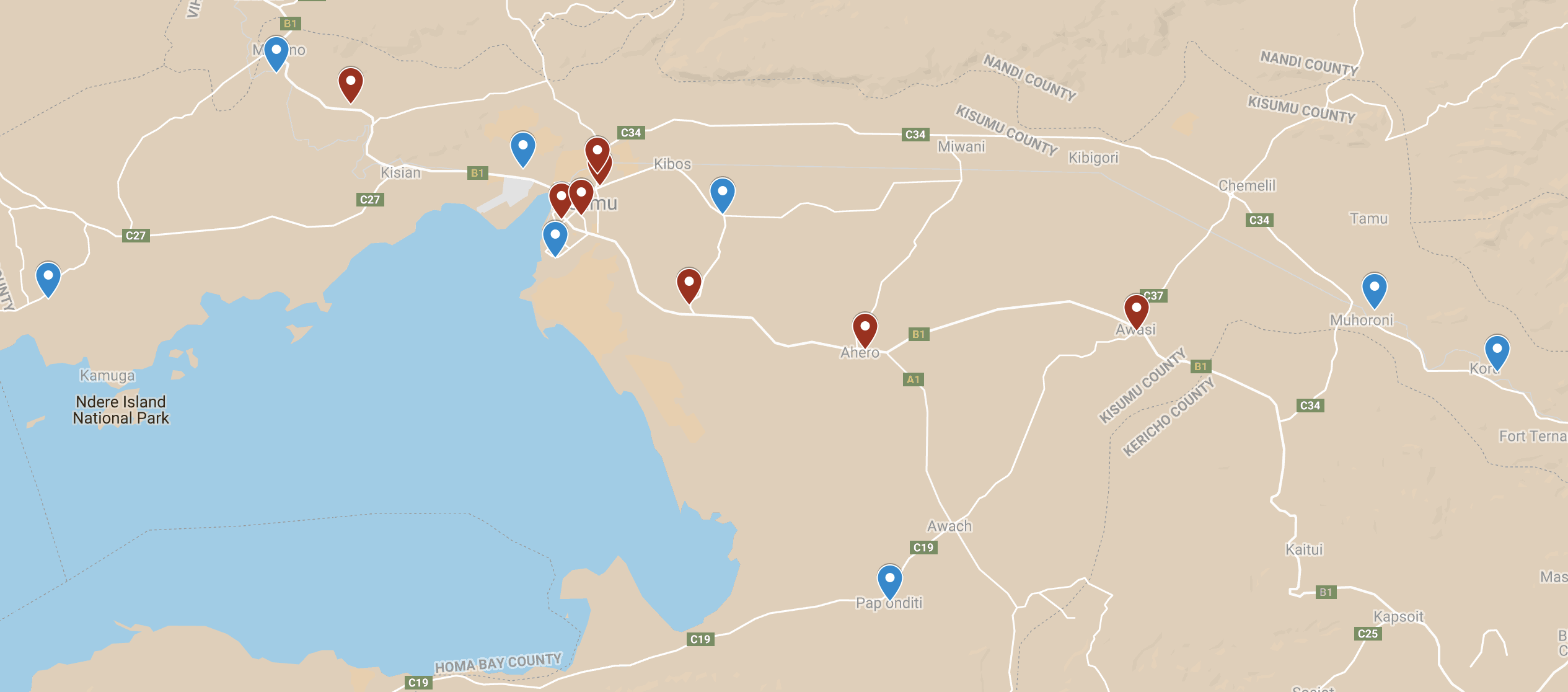
**

Red markers indicate facilities visited for time-and-motion studies

**E. Program Volume**

We used data collected as part of routine monitoring to estimate the numbers of women screened, initiated, and dispensed PrEP over a one-year period. Study staff abstracted standardized client records in all 16 facilities from November 20, 2017 to June 15, 2018. These records included behavioural risk assessment results for all clients counselled about PrEP and standard Ministry of Health records recording PrEP initiation and follow-up visits. Initiation and follow-up visit records included whether PrEP was dispensed as well as the next scheduled visit date. Out of 2586 follow-up visit records, 1963 (76%) indicated that PrEP was dispensed. We assumed that the remainder 24% of visits had no dispensation (regardless of whether dispensation status was recorded or missing).

To estimate annual output, we extrapolated the seven months of data to a full year (until November 19, 2018) assuming no temporal changes in volume. To do this, we created a synthetic cohort of clients entering the program between June 16, 2018 and November 19, 2018 assuming a pattern identical to the observed data starting November 20, 2017. However, the 248 clients who were continuing on PrEP as of June 15, 2018 were right censored. We assumed that right-censored clients who had fewer than two consecutive follow-up visits did not return for additional follow-ups, while right-censored clients who had at least two consecutive follow-up visits with recorded dispensation (post initiation) would continue PrEP until November 19, 2018. Our results were not sensitive to this assumption. Assuming instead that all right-censored clients never return to the clinic changes the cost per client-month of PrEP delivered only slightly (from $27 to $28).

To calculate the number of client-months of PrEP dispensed, we calculated the median time between recorded visits with PrEP dispensation and the next scheduled follow-up visit to be 28 days (interquartile range: 28-31). Therefore, we assumed that one month of PrEP were dispensed at each follow-up visit. As such, the total number of client-months of PrEP dispensed was calculated by summing the number of PrEP initiations and the number of PrEP follow-up visits with dispensation.

**F. MOH Scenario Results**

**Table S3: Total annual programme cost and unit cost per client-month of PrEP dispensed (2017 USD) in Ministry of Health (MOH) scenario^*^**

|  | **Total annual cost (USD)** | **Average cost per client-month of PrEP dispensed (USD)** |
| --- | --- | --- |
| ***Variable*** | | |
| Personnel (clinical) | 32,895 | 4.27 |
| Drugs | 51,997 | 6.75 |
| Laboratory testing | 18,560 | 2.41 |
| Other supplies | 3,616 | 0.47 |
| ***Sub-total*** | 107,068 | 13.90 |
| ***Fixed*** | | |
| Microplanning | 843 | 0.11 |
| Training | 1,978 | 0.26 |
| Personnel (supervision and administration) | 9,438 | 1.23 |
| Capital (e.g. creatinine machines, furniture) | 2,065 | 0.27 |
| Overhead (e.g. building, airtime, transportation) | 6,029 | 0.78 |
| ***Sub-total*** | 20,353 | 2.64 |
| **Summary** | **127,421** | **16.54** |

^*^The MOH scenario assumes public sector clinical staff salaries instead of study salaries; study administrative staff responsibilities are subsumed into routine facility, sub-county, and county supervision; and facility-based creatinine testing instead of point-of-care

**Table S4: Unit cost breakdown by clinical activity (2017 USD) under Ministry of Health (MOH) scenario^*^**

| **Cost by Clinical Activity (2017 USD)** |  |  |  |
| --- | --- | --- | --- |
|  | **Screening** | **Initiation** | **Follow-up**^†^ |
| *Variable unit cost* | | | |
| Personnel (clinical) | 0.80 | 1.29 | 1.87 |
| Drugs | 0.00 | 6.75 | 5.34 |
| Laboratory testing | 0.00 | 3.55 | 0.83 |
| Other supplies | 0.02 | 0.32 | 0.41 |
| ***Sub-total*** | **0.82** | **11.91** | **8.45** |
| *Fixed unit cost* | 0.43 | 1.37 | 0.97 |
| **Total unit cost (variable + fixed)** | **1.25** | **13.28** | **9.42** |
| Number | 24,005 | 4,198 | 4,427 |
| **Total annual cost** | **29,948** | **55,764** | **41,709** |

^†^Follow-up unit costs are weighted averages of visits with and without PrEP dispensation

^*^The MOH scenario assumes public sector clinical staff salaries instead of study salaries; study administrative staff responsibilities are subsumed into routine facility, sub-county, and county supervision; and facility-based creatinine testing instead of point-of-care

**Figure S3: Percentage of total program cost across cost categories as implemented and under Ministry of Health (MOH) scenario^*^**


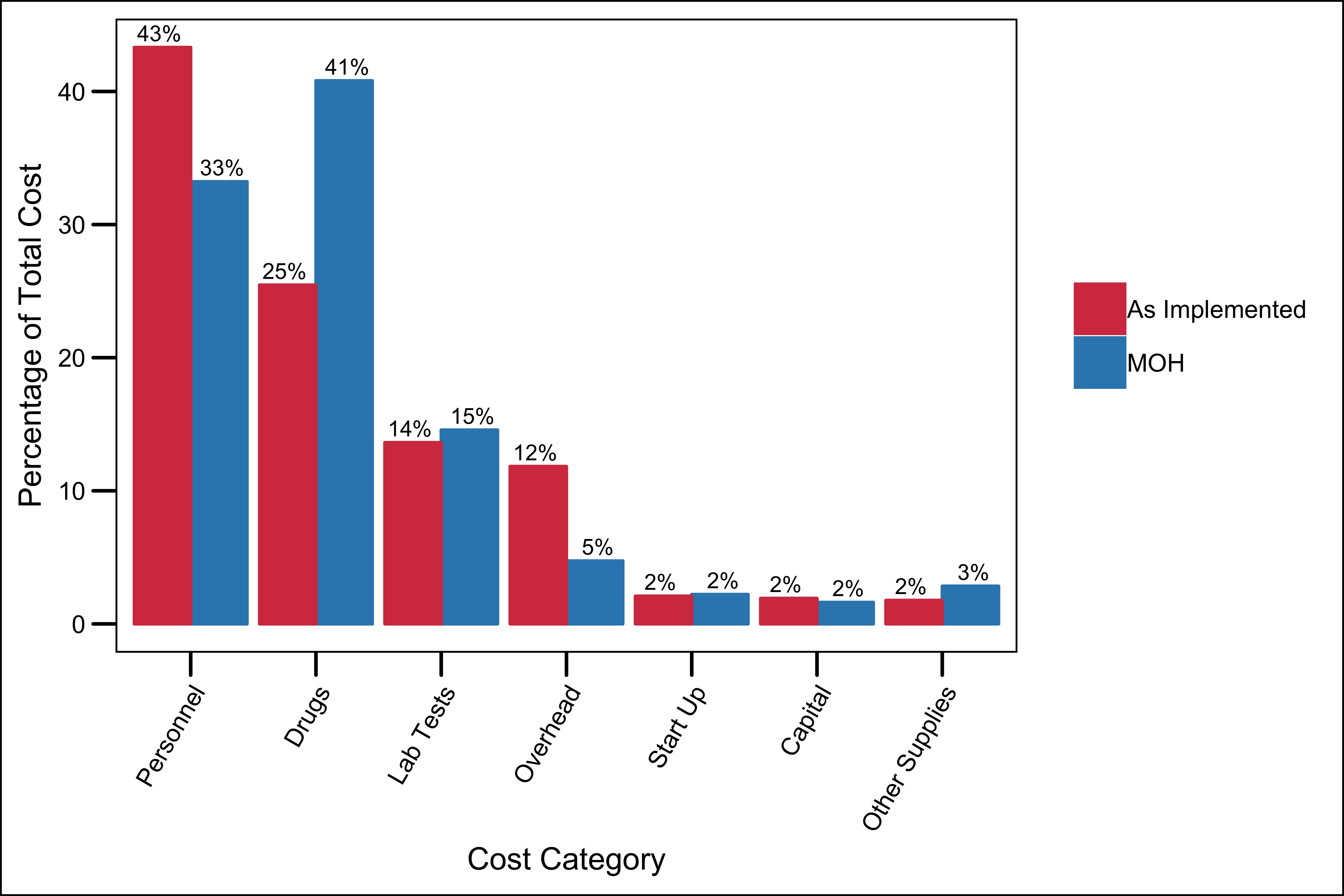


^*^The MOH scenario assumes public sector clinical staff salaries instead of study salaries; study administrative staff responsibilities are subsumed into routine facility, sub-county, and county supervision; and facility-based creatinine testing instead of point-of-care

**G. Sensitivity analysis of As Implemented costing results using different discount rates (2017 USD)**

| **Table S5: 5% Discount Rate** |  |  |
| --- | --- | --- |
|  | **Total annual cost (USD)** | **Average cost per client-month of PrEP dispensed (USD)** |
| ***Variable*** | | |
| Personnel (clinical) | 37,535 | 4.87 |
| Medication | 51,997 | 6.75 |
| Laboratory testing | 27,830 | 3.61 |
| Other supplies | 3,616 | 0.47 |
| ***Sub-total*** | 120,978 | 15.71 |
| ***Fixed*** | | |
| Microplanning | 1,445 | 0.19 |
| Training | 3,066 | 0.40 |
| Personnel (supervision and administration) | 50,924 | 6.61 |
| Capital (e.g. creatinine machines, furniture) | 3,987 | 0.52 |
| Overhead (e.g. building, airtime, transportation) | 24,162 | 3.14 |
| ***Sub-total*** | 83,584 | 10.85 |
| **Summary** | **204,562** | **26.56** |

| **Table S6: 10% Discount Rate** |  |  |
| --- | --- | --- |
|  | **Total annual cost**  **(USD)** | **Average cost per client-month of PrEP dispensed**  **(USD)** |
| ***Variable*** | | |
| Personnel (clinical) | 37,535 | 4.87 |
| Medication | 51,997 | 6.75 |
| Laboratory testing | 27,830 | 3.61 |
| Other supplies | 3,616 | 0.47 |
| ***Sub-total*** | 120,978 | 15.71 |
| ***Fixed*** | | |
| Microplanning | 1,650 | 0.21 |
| Training | 3,501 | 0.45 |
| Personnel (supervision and administration) | 50,924 | 6.61 |
| Capital (e.g. creatinine machines, furniture) | 4,149 | 0.54 |
| Overhead (e.g. building, airtime, transportation) | 24,162 | 3.14 |
| ***Sub-total*** | 84,387 | 10.96 |
| **Summary** | **205,365** | **26.66** |

| **Table S7: 15% Discount Rate** |  |  |
| --- | --- | --- |
|  | **Total annual cost**  **(USD)** | **Average cost per client-month of PrEP dispensed**  **(USD)** |
| ***Variable*** | | |
| Personnel (clinical) | 37,535 | 4.87 |
| Medication | 51,997 | 6.75 |
| Laboratory testing | 27,830 | 3.61 |
| Other supplies | 3,616 | 0.47 |
| ***Sub-total*** | 120,978 | 15.71 |
| ***Fixed*** | | |
| Microplanning | 1,866 | 0.24 |
| Training | 3,959 | 0.51 |
| Personnel (supervision and administration) | 50,924 | 6.61 |
| Capital (e.g. creatinine machines, furniture) | 4,319 | 0.56 |
| Overhead (e.g. building, airtime, transportation) | 24,162 | 3.14 |
| ***Sub-total*** | 85,231 | 11.07 |
| **Summary** | **206,209** | **26.77** |
